# How can we measure the developmental impacts of the COVID-19 pandemic on young children?

**DOI:** 10.23889/ijpds.v7i3.1803

**Published:** 2022-08-25

**Authors:** Amy Hawn Nelson, Emily Berkowitz

**Affiliations:** 1 Actionable Intelligence for Social Policy (AISP), University of Pennsylvania School of Social Policy & Practice

## Objectives

- Present a conceptual data model for understanding the impacts of COVID on children 0-5
- Outline administrative datasets with relevant indicators that are commonly collected, integrated at the individual level, and include relevant 2-generation linkages
- Provide specific considerations for research using the conceptual model

## Approach

This session presents findings from a paper that was collaboratively generated by a workgroup of five integrated administrative data systems (IDS) in communities across the US with expansive data holdings in early education. Contributors have decades of experience in utilizing administrative data for social policy planning and analysis. We started with a thorough literature review, used a national survey data of IDS data holdings collected in 2020, and co-created a conceptual data model for administrative data reuse based upon current practices. Specific care was taken to include community-level changes and adult factors that serve as mediators for children’s outcomes.

## Results

The COVID-19 pandemic made its mark on the entire world, yet little is known about children, aged 0-5, whose lives have been exponentially affected. This conceptual data model presents five developmental resilience pathways (i.e. early learning, safe and nurturing families, health, housing, and financial/employment) that include direct and indirect influencers related to COVID-19 impacts, and the contexts and community supports that can affect child-level outcomes. We then overview commonly available administrative datasets with relevant indicators to be linked at the individual and household level, with discussion of data specific considerations (e.g. access, insufficiency, availability, quality, and linkage). The US response creates a natural experiment of policy and implementation decisions across boundaries, and while US-centric, this approach could be applied internationally.

## Conclusion

The development and use of IDS for research has great potential to inform solutions to our most pressing social problems. Cross-site research studies and maintaining sustainable capacities for long-term research is paramount to understanding the implications of response interventions and to study impacts on child development over time.

